# Craving and personal functioning as mediators of extended‐release buprenorphine efficacy: A four‐way decomposition analysis from a randomised trial

**DOI:** 10.1111/add.70521

**Published:** 2026-07-01

**Authors:** John Marsden, Mike Kelleher, Fiona Cowden, Edward Day, Jonathan Dewhurst, Rachel Evans, Eilish Gilvarry, Natalie Lowry, Luke Mitcheson, Robert Murray, Sophie Quarshie, Zoë Hoare

**Affiliations:** ^1^ Addictions Department, School of Academic Psychiatry, Institute of Psychiatry, Psychology and Neuroscience King's College London London UK; ^2^ South London and Maudsley NHS Foundation Trust London UK; ^3^ NHS Tayside and Dundee Health and Social Care Partnership Dundee UK; ^4^ Birmingham and Solihull Mental Health NHS Foundation Trust Birmingham UK; ^5^ Greater Manchester Mental Health NHS Foundation Trust Manchester UK; ^6^ North Wales Medical School, Bangor University Bangor Gwynedd UK; ^7^ Cumbria, Northumberland, Tyne and Wear NHS Foundation Trust Newcastle Upon Tyne UK

**Keywords:** causal mediation, extended‐release buprenorphine, four‐way decomposition, opioid use disorder, randomised controlled trial, secondary analysis

## Abstract

**Aims:**

To investigate whether the treatment effect of extended‐release buprenorphine (BUP‐XR) on opioid‐free days in opioid use disorder (OUD) is mediated by reductions in opioid craving and improvements in personal functioning.

**Design and setting:**

Exploratory causal mediation analysis using VanderWeele's four‐way decomposition framework in a 24‐week, multi‐centre, open‐label, superiority, randomised controlled trial comparing methadone or sublingual buprenorphine (MET/BUP‐SL) with extended‐release buprenorphine (BUP‐XR).

**Participants:**

Adults with *Diagnostic and Statistical Manual of Mental Disorders, Fifth Edition* moderate to severe OUD (*n* = 314; 98% severe). Mean age was 42 years, 32% were female and 44% were using non‐medical opioids, predominantly heroin, at study baseline.

**Intervention and comparator:**

MET/BUP‐SL vs. BUP‐XR (1:1 randomisation).

**Measurements:**

The outcome was the number of opioid‐free days (0–84) during weeks 13–24. Candidate mediators were visual analogue scale ratings of opioid ‘need’ and ‘want’ (VAS‐N, VAS‐W), craving imagery and intrusiveness [Craving Experiences Questionnaire – frequency version (CEQ‐F)] and personal functioning impairment [Work and Social Adjustment Scale (WSAS)]. Craving measures were assessed at baseline and weeks 4, 8 and 12, then reduced to binary summaries indicating craving absent at all follow‐up assessments vs. present at any assessment. WSAS was assessed at week 12 and retained as a continuous mediator.

**Findings:**

During weeks 13–24, the adjusted mean number of opioid‐free days was 48.9 in the MET/BUP‐SL group and 58.5 in the BUP‐XR group [incidence rate ratio (IRR) = 1.20, 95% confidence interval (CI) = 1.10–1.30; *P*‐value <0.001]. Across the four mediator‐specific models, BUP‐XR was associated with more opioid‐free days (total‐effect IRRs = 1.10–1.18; all *P*‐values <0.05). BUP‐XR increased the odds of craving absence for VAS‐N and VAS‐W. VAS‐N was the only mediator showing evidence of indirect mediation [pure indirect effect (PIE) = 1.05, 95% CI = 1.01–1.10; *P*‐value = 0.015], indicating that reduced opioid ‘need’ contributed to the treatment effect. CEQ‐F and VAS‐W showed small, non‐statistically significant indirect effects. BUP‐XR improved personal functioning (WSAS mean difference −3.79; *P*‐value = 0.014), although WSAS showed no clear evidence of mediation (PIE = 1.05; *P*‐value = 0.085). Findings were broadly consistent across deterministic and multiple imputation sensitivity analyses, with VAS‐N remaining the clearest candidate mediator.

**Conclusions:**

In a pre‐registered exploratory secondary analysis of a randomised controlled trial, the beneficial effect of extended‐release buprenorphine among people with opioid use disorder (OUD) on opioid‐free days appeared to operate mainly through direct pathways, with additional evidence that reduced need‐related craving contributed to benefit. Improved personal functioning occurred alongside, rather than clearly mediating, treatment benefit. Need‐related craving may represent a useful proximal mechanistic endpoint in OUD pharmacotherapy trials.

## INTRODUCTION

Opioid use disorder (OUD) is characterised by compulsive opioid use despite harms to health and personal functioning [1]. Early opioid use is reinforced by activation of the μ‐opioid receptor (μ‐OR) system and associated reward pathways. With repeated use, opioid‐related cues can acquire motivational salience [2]. Stress‐system dysregulation then increasingly motivates opioid use to alleviate dysphoria and restore physiological normality [3, 4].

Craving is a central clinical and psychological feature of OUD [5]. It reflects conditioned incentive processes and reduced inhibitory control [6]. OUD is also associated with a range of health and social harms, including mood disturbance [7], strained interpersonal relationships [8], social withdrawal [9], and unemployment [10].

The international standard of care for OUD is maintenance pharmacotherapy with oral methadone (MET) (a full μ‐OR agonist; recommended dose range 60–120 mg/day) or sublingual buprenorphine (BUP‐SL) (a partial μ‐OR agonist; recommended dose range 8–24 mg/day) [11, 12]. MET and BUP‐SL provide sustained μ‐OR occupancy, prevent withdrawal, and reduce the reinforcing effects of non‐medical opioid use [13, 14]. Maintenance treatment offers a platform for psychosocial support and medical care [15]. However, many patients continue to use non‐medical opioids or discontinue treatment. In a systematic review of 67 cohort studies, the median 1‐year retention rate was 57% [16]. Approximately 40% to 50% of patients who leave MET and BUP‐SL treatment relapse within 6 months [17].

Extended‐release buprenorphine (BUP‐XR), administered subcutaneously, was developed to overcome limitations of daily treatment by offering sustained suppression of withdrawal symptoms and blockade of the reinforcing effects of non‐medical opioids [18]. Two formulations are approved: Buvidal [weekly and monthly; Brixadi in the United States (US)] and Sublocade (monthly).

The Extended‐Release Pharmacotherapy for OUD (EXPO) trial was the first multi‐site, open‐label, superiority, randomised controlled trial (RCT) comparing MET/BUP‐SL (*n* = 156) with BUP‐XR (*n* = 158). BUP‐XR was associated with more opioid‐free days, longer time in study treatment (mean retention 128.5 days in the MET/BUP‐SL group and 144.6 days in the BUP‐XR group), improved personal functioning and reduced opioid craving [19]. Follow‐up craving scores showed a bimodal pattern of an increasing proportion of participants reporting complete absence of craving, whereas others continued to report craving.

EXPO included exploratory subgroup analyses based on baseline clinician‐rated OUD severity on the Clinical Global Impression–Severity scale (CGI‐S) [20], benzodiazepine use in the past month and duration in maintenance treatment at enrolment (<28 days versus ≥28 days). The relative benefit of BUP‐XR appeared greater among participants with more severe OUD and among those reporting benzodiazepine use, suggesting that treatment benefit was not uniform across the study population.

We hypothesised that the benefit of BUP‐XR is partly mediated by reductions in craving and partly by improvements in personal functioning. These pathways can be examined using causal mediation analysis. In an RCT, random allocation removes confounding of the treatment–mediator and treatment–outcome associations, and temporal ordering is established when the mediator is measured after treatment initiation and before the outcome. However, because the mediator is not randomised, valid identification still requires adjustment for pre‐treatment confounders of the mediator–outcome association and avoidance of conditioning on post‐treatment variables affected by treatment [21].

Despite extensive clinical trial evidence for MET and BUP‐SL efficacy, treatment mechanisms have rarely been examined. To our knowledge, only one published trial analysis has evaluated craving as a mediator of buprenorphine‐based treatment effects on opioid use, estimating an average indirect effect [22].

The EXPO protocol pre‐specified an exploratory causal‐inference analysis to estimate the contribution of candidate mediators consistent with hypothesised mechanisms of BUP‐XR efficacy [23]. The present study had three aims: (1) to estimate the extent to which the effect of BUP‐XR versus daily oral treatment on opioid‐free days during weeks 13 to 24 was mediated by opioid ‘need’, opioid ‘want’, craving imagery/frequency and personal functioning; (2) to assess whether any mediated effects involved treatment–mediator interaction; and (3) to examine whether these effects were modified by baseline OUD severity and duration of treatment before enrolment.

## METHOD

### Design and participants

This was an exploratory counterfactual mediation analysis using four‐way decomposition of data from the EXPO multi‐site, open‐label, superiority RCT (recruitment: August 2019–November 2021; final follow‐up: April 2022). The analysis was reported in accordance with the A Guideline for Reporting Mediation Analyses‐Short Form (AGReMA‐SF) statement [24].

Participants were recruited from National Health Service (NHS) community addiction treatment services in England and Scotland. Eligible participants were adults 18 years old and older with Diagnostic and Statistical Manual of Mental Disorders, Fifth Edition (DSM‐5) moderate‐to‐severe OUD at admission to their current standard‐of‐care maintenance treatment episode. At screening, participants were receiving MET or BUP‐SL within protocol dose thresholds. Recruitment reflected routine treatment pathways in which individuals were either already engaged in, or newly admitted to, opioid agonist treatment within community services.

### Screening and treatment allocation

For participants entering the trial from MET, eligibility for screening required a dose of ≤50 mg/day. Patients receiving higher MET doses who wished to participate were offered a taper to the screening threshold. Following protocol amendment, the screening threshold was reduced to ≤30 mg/day. This ensured that, if allocated to BUP‐XR, participants could complete a BUP‐SL run‐in within 7 days.

Participants were randomly allocated (1:1) to 24 weeks of daily oral treatment with MET or BUP‐SL (coded 0) or to BUP‐XR (coded 1). Randomisation itself did not require a change in current medication. Doses of MET and BUP‐SL were determined by the treating clinic in accordance with United Kingdom (UK) clinical guidelines. BUP‐XR (Sublocade) was administered as a 300 mg loading dose followed by four monthly injections of 100 mg or 300 mg, with maintenance doses selected according to clinical response and patient preference.

During the trial, 155 (99.4%) of 156 participants allocated to MET/BUP‐SL received at least one dose of their allocated medication. Available maintenance‐dose data indicated a mean MET dose of 28.9 mg/day (range, 20–61 mg/day) and a mean BUP‐SL dose of 12.0 mg/day (range, 1–26 mg/day). In the BUP‐XR treatment group, 150 (94.9%) of 158 participants received at least one dose of their allocated study drug, with participants receiving a mean of 5 of 6 scheduled injections.

### Outcome measure

The outcome was the number of non‐medical opioid‐free days during follow‐up weeks 13 to 24. This 84‐day window was selected to preserve temporal ordering, such that mediator assessment, completed by week 12, preceded the outcome period. Each day was classified as opioid‐free or not opioid‐free using data from fortnightly clinic visit‐administered Timeline Followback (TLFB) interviews and point‐of‐care urine drug screens (UDS), supplemented by information from participants' electronic health records where required. Days with neither self‐reported nor biologically indicated non‐medical opioid use were classified as opioid‐free. Assuming a 3‐day detection window, a positive UDS was recorded in the TLFB as opioid use on the day of testing and the two preceding days.

### Mediators

The EXPO protocol specified standardised instruments assessing craving, neurocognitive status, depressive symptoms and personal functioning as candidate mediators. The Montreal Cognitive Assessment [25] was discontinued during fieldwork because of the coronavirus disease 2019 restrictions on in‐person testing. Depressive symptoms were assessed using the Quick Inventory of Depressive Symptomatology‐Self‐Report [26]. In the report on the primary findings, depressive symptoms showed no evidence of a treatment effect [19], so this measure was not considered further for mediation analysis as identification of mediation requires evidence of an association between treatment and mediator.

The remaining four mediators were retained because they were pre‐specified, conceptually relevant candidate mechanisms and showed either evidence of treatment effect in the parent trial or sufficient relevance to the hypothesised pathways to warrant exploratory decomposition:

1. Visual analogue scale (0–100 mm) rating of maximum perceived ‘need’ for opioids in the past 2 weeks (VAS‐N) [27];
2. Visual analogue scale (0–100 mm) rating of maximum perceived ‘want’ for opioids in the past 2 weeks (VAS‐W) [27];
3. The Craving Experiences Questionnaire–Frequency version (CEQ‐F) [28], an 11‐item measure of the frequency, intensity and intrusiveness of mental imagery‐related aspects of opioid craving in the past 2 weeks (Cronbach's α = 0.83 in a clinical sample; score range 0–110, with higher scores indicating greater craving); and
4. The Work and Social Adjustment Scale (WSAS) [29], a five‐item measure of personal functioning impairment in work, home management, social and private leisure activities and close relationships in the past 2 weeks (score range 0–40, higher scores indicating greater impairment).

VAS‐N, VAS‐W and CEQ‐F were analysed as separate mediators because they capture related but distinct aspects of craving: perceived opioid ‘need’, perceived opioid ‘want’ and the frequency, intensity and intrusiveness of craving‐related mental imagery. These measures were completed at baseline and at weeks 4, 8 and 12. In the primary EXPO analysis, follow‐up distributions were bimodal, with an increasing proportion of participants reporting no craving. We, therefore, derived a binary summary for each craving‐related mediator across weeks 4, 8 and 12. Participants were coded 1 (craving absent) if they reported a score of 0 at all three assessments, and 0 (craving present) if they reported any non‐zero score at any assessment.

The WSAS was taken from the week 12 assessment and retained as a continuous mediator because its follow‐up distribution did not show the same zero‐inflated pattern as the craving measures.

### Treatment‐effect modifiers

Individual‐level treatment‐effect modifiers were baseline clinician‐rated OUD severity using the CGI‐S, dichotomised as moderate to severe (coded 1) versus less than moderate (coded 0), and the duration of the current treatment episode before EXPO enrolment (<28 days versus ≥28 days). Although baseline benzodiazepine use was associated with BUP‐XR benefit in the primary EXPO analysis, the number of participants reporting benzodiazepine use (72/314; 22.9%) was considered insufficient to support reliable estimation of interaction effects in the present analysis and was, therefore, not included.

### Statistical analysis

Analyses were conducted in Stata (v18) using the *med4way* command. This estimates the total effect and implements VanderWeele's four‐way decomposition (Figure 1):

1. Controlled direct effect (CDE), which represents the treatment effect on outcome when the mediator is fixed at a specified level;
2. Pure indirect effect (PIE), which represents the component of the treatment effect transmitted through the mediator alone, excluding any contribution from treatment–mediator interaction;
3. Reference interaction (INTref), which represents the treatment × mediator interaction when the mediator is fixed at its reference level; and
4. Mediated interaction (INTmed), which represents the component arising jointly from treatment changing the mediator and treatment–mediator interaction.

**FIGURE 1:**
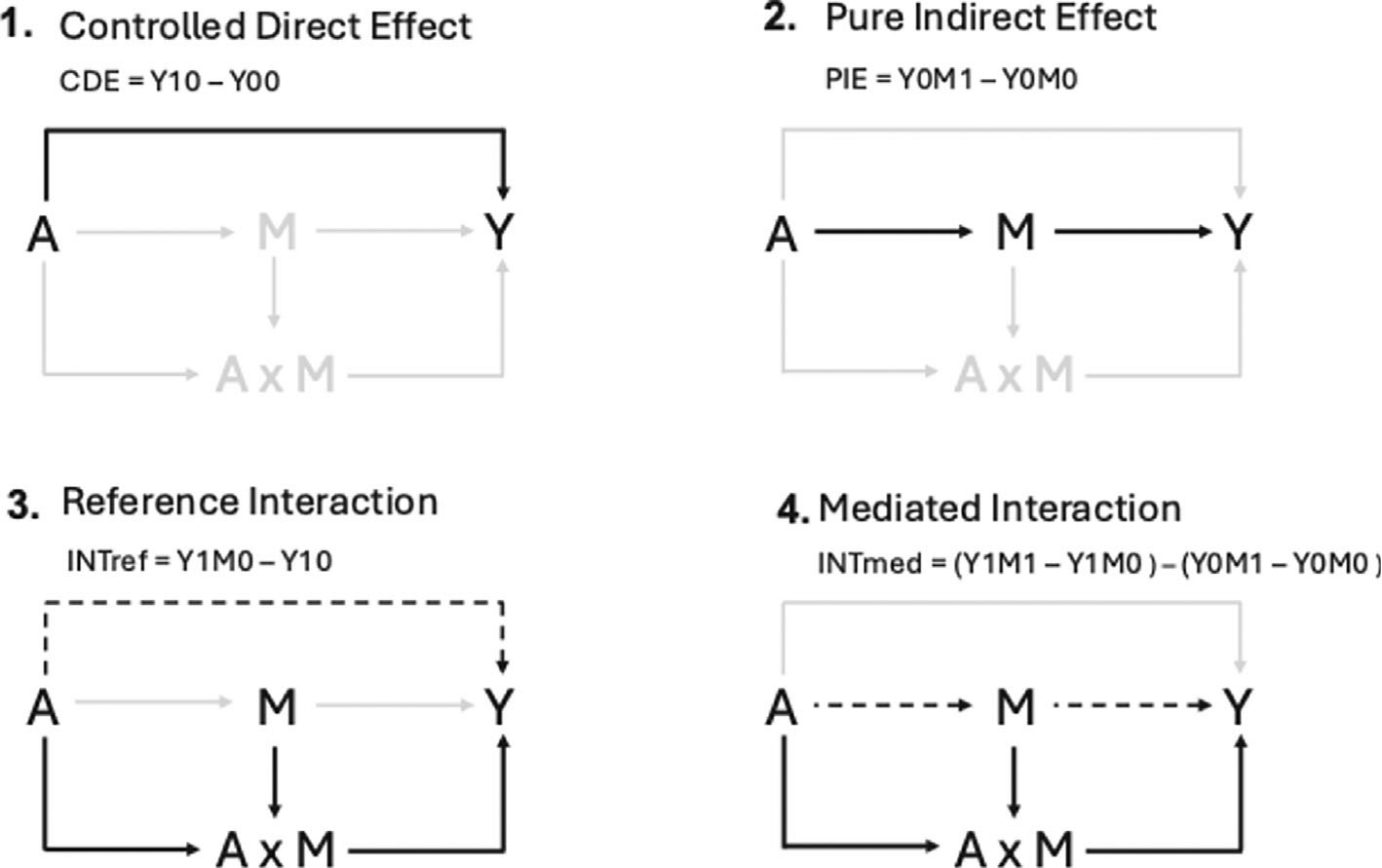
Schematic for a four‐way decomposition. Where (for the binary coded craving mediators): a, randomly allocated treatment [0 = methadone/ sublingual buprenorphine (MET/BUP‐SL); 1 = extended‐release buprenorphine (BUP‐XR)]; M, mediator (0 = present; 1 = absent); M0, value the mediator would take under MET/BUP‐SL (a = 0); M1, value the mediator would take under BUP‐XR (a = 1); a × M, treatment–mediator interaction; Y, outcome (count of opioid‐free days during weeks 13–24); Y10 and Y00, outcomes under BUP‐XR and MET/BUP‐SL, respectively, when the mediator is fixed at M = 0; Y1M0 and Y0M0, outcomes under BUP‐XR and MET/BUP‐SL, respectively, when the mediator takes the value it would under MET/BUP‐SL; Y1M1 and Y0M1, outcomes under BUP‐XR and MET/BUP‐SL, respectively, when the mediator takes the value it would under BUP‐XR. The controlled direct effect (CDE) represents the effect of treatment on the outcome when the mediator is held fixed at the reference level. The pure indirect effect (PIE) represents the effect operating through changes in the mediator in the absence of treatment–mediator interaction. The reference interaction (INTref) represents the effect attributable to treatment–mediator interaction alone, with the mediator fixed at the reference level. The mediated interaction (INTmed) represents the joint effect of mediation and interaction, whereby treatment changes the mediator and the mediator modifies the treatment effect on the outcome. INTmed <1 indicates antagonistic mediated interaction; INTmed >1 indicates synergistic mediated interaction (on the multiplicative scale). Note: WSAS was analysed on its continuous scale; estimates, therefore, reflect the observed distribution of mediator values rather than binary contrasts.

The total effect and four decomposition components were expressed as adjusted incidence rate ratios (IRRs), with IRR values >1 indicating more opioid‐free days and IRR values <1 indicating fewer opioid‐free days. For the craving‐related mediators, the reference level was craving present [mediator (M) = 0] and the comparison level was craving absent (M = 1); CDE and INTref were interpreted relative to this coding. For the interaction components (INTref and INTmed), IRR values <1 indicate antagonistic interaction (the joint effect of treatment and mediator change is smaller than expected), whereas values >1 indicate synergistic interaction (the joint effect is larger than expected).

The mediator models for binary measures, VAS‐N, VAS‐W and CEQ‐F, were specified using logistic regression. For WSAS, a continuous measure, the mediator model was specified using linear regression. The outcome model was specified as negative binomial regression analysing the outcome as a count variable and included terms for treatment, mediator and the treatment × mediator interaction.

All mediator and outcome models were adjusted for two pre‐treatment covariates: (1) baseline heroin use (participant‐reported heroin use on ≥1 day in the 28 days before random allocation), included because recent heroin use may influence both craving and subsequent opioid use, and (2) the baseline value of the candidate mediator, included to account for pre‐treatment level of the construct and reduce potential mediator–outcome confounding [30]. Participant age and sex were examined as additional covariates, but were not retained because they did not materially alter model fit or effect estimates.

The analysis was done in three stages. First, we fitted adjusted mediator models to examine the association between treatment allocation and each mediator. Second, we fitted adjusted models for opioid‐free days during weeks 13 to 24 including treatment group, baseline covariates, mediator and a treatment × mediator interaction term. A relatively small proportion of participants (*n* = 51, 16%) provided no TLFB data during weeks 13 to 24. For these individuals, 1 day of non‐abstinence was assigned, consistent with the parent EXPO analysis. This approach preserved inclusion in the count model while avoiding the assumption of complete abstinence during unobserved periods. An exposure variable representing the amount of observed TLFB/UDS follow‐up time was incorporated into the negative binomial outcome model to account for differences in observation time, allowing all available information to be retained.

To assess the impact of missing data on baseline variables (Table 1), missing continuous baseline variables were imputed using the sample mean, and missing baseline heroin use data was conservatively coded as heroin use present. Deterministic sensitivity analyses were then conducted under best‐case and worst‐case assumptions. For craving‐related mediators, missing values were coded as craving present under the worst‐case scenario and craving absent under the best‐case scenario, with analyses repeated under each assumption. For WSAS, missing week 12 values were replaced with the mean within the corresponding treatment group, and analyses were repeated.

**TABLE 1:** Participant characteristics and baseline values of mediators at follow‐up.

Characteristic	MET/BUP‐SL (*n* = 156)	BUP‐XR (*n* = 158)	Overall (*n* = 314)
Participant‐demographic			
Age‐years	41.6 (8.2)	42.5 (7.9)	42.0 (8.1)
Sex‐assigned at birth			
Male	111 (71.2)	122 (77.2)	233 (74.2)
Female	45 (28.9)	36 (22.8)	81 (25.8)
Participant‐clinical characteristics			
Mean age first enrolled in MET/BUP‐SL (SD)	29.8 (9.3)	30.4 (8.8)	30.1 (9.0)
Duration of treatment episode at enrolment mean weeks (range)	39.8 (2.4–167.3)	58.9 (4.0–183.3)	49.9 (3.0–176.6)
Baseline measures			
CGI‐S			
Extremely mild–mild	90 (57.7)	90 (57.0)	180 (57.3)
Moderate–extreme	66 (42.3)	68 (43.0)	134 (42.7)
Drug use, past 28 days			
Heroin^a^	74 (47.4)	64 (40.5)	138 (44.0)
CEQ‐F, mean^b^	29.9 (34.3)	27.9 (33.2)	28.9 (33.7)
VAS‐N, mean^c^	27.4 (39.4)	25.8 (38.4)	26.6 (38.8)
VAS‐W, mean^c^	33.5 (38.9)	31.0 (36.3)	32.2 (37.6)
WSAS, mean^c^	16.9 (12.8)	16.1 (13.6)	16.5 (13.2)
Mediators at follow‐up	(*n* = 123)	(*n* = 147)	(*n* = 270)
CEQ‐F^d^			
Present	85 (69.1)	79 (53.7)	164 (60.7)
Absent	38 (30.8)	68 (46.3)	106 (39.3)
VAS‐N^d^			
Present	63 (51.2)	38 (25.9)	101 (37.4)
Absent	60 (48.8)	109 (74.1)	169 (62.6)
VAS‐W^d^			
Present	80 (65.0)	65 (44.2)	145 (53.7)
Absent	43 (35.0)	82 (55.8)	125 (46.3)
WSAS^e^			
Mean	14.1 (12.8)	8.2 (11.8)	10.6 (12.5)

*Note*: Data are number (percentage) unless otherwise stated.

Abbreviations: BUP‐XR, extended‐release buprenorphine; CEQ‐F, Craving Experiences Questionnaire‐Frequency version; CGI‐S, Clinical Global Impression‐Severity scale; MET/BUP‐SL, methadone or sublingual buprenorphine; VAS‐N, Visual Analogue Scale for maximum ‘need’ for opioids; VAS‐W, Visual Analogue Scale for maximum ‘want’ for opioids; SD, standard deviation; WSAS, Work and Social Adjustment Scale.

^a^ Missing data for 24 participants (8 in MET/BUP‐SL, 16 in BUP‐XR).

^b^ Missing data for 2 participants in BUP‐XR.

^c^ Missing data for 1 participant in BUP‐XR.

^d^ Missing data for 44 participants (33 in MET/BUP‐SL, 11 in BUP‐XR).

^e^ Missing data for 96 participants (67 in MET/BUP‐SL, 29 in BUP‐XR).

Additional sensitivity analyses were conducted using multiple imputation by chained equations (MICE) for intermittent missing baseline and mediator data under missing‐at‐random assumptions. Multiple imputation models (Stata command: mi impute chained) generated 50 imputed datasets, which included treatment allocation, baseline heroin use, baseline value of the candidate mediator, age and sex. Logistic regression imputation was used for binary craving mediators (CEQ‐F, VAS‐N and VAS‐W), and predictive mean matching was used for the WSAS. Primary regression models were repeated across imputed datasets, with estimates combined using Rubin's rules [31].

The *med4way* package does not support pooling bootstrap‐based decomposition estimates across multiply imputed datasets, therefore, the MICE analyses were used to assess the robustness of the mediator and outcome models underlying the decomposition rather than to re‐estimate the four‐way decomposition itself. Results were compared with the complete‐case and deterministic sensitivity analyses.

Third, we used *med4way* to combine the specified mediator and outcome models and estimate the total effect and the four decomposition components. Uncertainty was quantified using non‐parametric bootstrap resampling of participants with replacement (1000 replicates). For each bootstrap replicate, the mediator and outcome models were refitted, and the total effect and four decomposition components were recomputed. Bootstrap‐derived 95% CIs and two‐sided *P*‐values were estimated, with α = 0.05.

Robustness of the total treatment effect was examined using the E‐value, which quantifies the minimum strength of association that an unmeasured factor would need to have with both the treatment and the outcome, conditional on measured covariates, to fully explain away the observed association [32]. E‐values were calculated for the total‐effect IRR point estimate and for the confidence limit closest to the null. Finally, we examined treatment‐effect modification by repeating the mediation analyses within strata defined by baseline OUD severity (CGI‐S) and duration of treatment before enrolment.

## RESULTS

A total of 314 participants were randomised to MET/BUP‐SL (*n* = 156) or BUP‐XR (*n* = 158). A total of 210 participants (66.9%) completed the study: 54.5% in the MET/BUP‐SL group and 79.1% in the BUP‐XR group. The treatment groups were broadly similar at baseline with respect to demographic characteristics, drug use and mediator scores (Table 1).

### Treatment effect

During follow‐up weeks 13 to 24, the adjusted mean number of opioid‐free days was 48.9 (95% CI = 44.6–53.1) in the MET/BUP‐SL group and 58.5 (95% CI = 56.4–60.5) in the BUP‐XR group. This corresponded to a total treatment effect IRR of 1.20 (95% CI = 1.10–1.30; *P*‐value <0.001).

### Mediator and outcome models

Table 2 presents the adjusted mediator (treatment–mediator) and outcome (mediator–outcome) models used to inform the mediation analysis. In the adjusted mediator models, baseline mediator values were strong predictors of their corresponding follow‐up measures. Across the craving measures, baseline heroin use was associated with lower odds of craving absence at follow‐up, but was not associated with opioid‐free days in the corresponding outcome models.

**TABLE 2:** Adjusted mediator and outcome models for BUP‐XR versus MET/BUP‐SL.

Measure	Model	Analysis	Effect	95% CI	*P*‐value
CEQ‐F (*n* = 246)	OR	Treatment group	1.65	0.90–3.01	0.104
		Baseline heroin use	0.32	0.16–0.65	0.002
		Baseline craving	0.97	0.96–0.99	<0.001
	IRR	Treatment group	1.25	1.12–1.41	<0.001
		CEQ‐F	1.18	1.04–1.34	0.010
		Treatment group × CEQ‐F	0.83	0.73–0.96	0.009
		Baseline heroin use	0.95	0.86–1.04	0.261
		Baseline craving	0.997	0.99–0.99	0.007
VAS‐N (*n* = 247)	OR	Treatment group	2.83	1.57–5.10	0.001
		Baseline heroin	0.28	0.15–0.54	<0.001
		Baseline craving	0.99	0.98–0.99	0.004
	IRR	Treatment group	1.38	1.17–1.63	<0.001
		VAS‐N	1.35	1.16–1.58	<0.001
		Treatment group × VAS‐N	0.74	0.62–0.89	0.001
		Baseline heroin use	0.95	0.87–1.04	0.291
		Baseline craving	0.99	0.99–0.99	0.008
VAS‐W (*n* = 247)	OR	Treatment group	2.39	1.30–4.38	0.005
		Baseline heroin use	0.30	0.15–0.60	0.001
		Baseline craving	0.98	0.97–0.99	<0.001
	IRR	Treatment group	1.23	1.09–1.39	0.001
		VAS‐W	1.12	0.94–1.31	0.182
		Treatment group × VAS‐W	0.89	0.76–1.03	0.133
		Baseline heroin use	0.93	0.85–1.02	0.125
		Baseline craving	0.99	0.99–0.99	0.027
WSAS (*n* = 196)	Coef.	Treatment group	−3.79	−6.82 to −0.77	0.014
		Baseline heroin use	3.99	0.82–7.15	0.013
		Baseline WSAS	0.38	0.26–0.50	<0.001
	IRR	Treatment group	0.97	0.91–1.04	0.353
		WSAS	0.99	0.98–0.99	0.001
		Treatment group × WSAS	1.01	1.00–1.02	0.004
		Baseline heroin use	0.88	0.83–0.93	<0.001
		Baseline WSAS	0.99	0.99–0.99	0.046

*Note*: Treatment group (0, MET/BUP‐SL; 1, BUP‐XR),

OR and Coef. rows show adjusted mediator models for craving (logistic regression) and personal functioning (linear regression). IRR rows show adjusted negative binomial outcome models for opioid‐free days including treatment group, mediator, baseline covariates and the treatment × mediator interaction.

Abbreviations: BUP‐XR, extended‐release buprenorphine; CEQ‐F, Craving Experiences Questionnaire‐Frequency version; Coef., coefficient; IRR, adjusted Incidence Rate Ratio; MET/BUP‐SL, methadone or sublingual buprenorphine; OR, adjusted odds ratio; OUD, opioid use disorder; VAS‐N, ‘need’ for opioids; VAS‐W, ‘want’ for opioids; WSAS, Work and Social Adjustment Scale (for OUD).

Consistent with the criteria for mediation analysis, evidence of a treatment effect on candidate mediators was assessed in these models. BUP‐XR was associated with greater odds of craving absence for VAS‐N (OR = 2.83, 95% CI = 1.57–5.10, *P*‐value = 0.001) and VAS‐W (OR = 2.39, 95% CI = 1.30–4.38, *P*‐value = 0.005), whereas the association for CEQ‐F was in the same direction, but was not statistically significant (OR = 1.65, 95% CI = 0.90–3.01, *P*‐value = 0.104).

In the outcome models, craving absence was associated with more opioid‐free days for VAS‐N (IRR = 1.35, 95% CI = 1.16–1.58, *P*‐value = <0.001) and CEQ‐F (IRR = 1.18, 95% CI = 1.04–1.34, *P*‐value = 0.010), whereas VAS‐W showed a weaker, non‐statistically significant association with opioid‐free days (IRR = 1.12, 95% CI = 0.94–1.31, *P*‐value = 0.182). Treatment × mediator interaction terms were <1 for all three craving measures, indicating antagonistic interaction on the multiplicative scale. This was strongest for VAS‐N (IRR = 0.74, 95% CI = 0.62–0.89, *P*‐value = 0.001), weaker for CEQ‐F (IRR = 0.83, 95% CI = 0.73–0.96, *P*‐value = 0.009) and not statistically significant for VAS‐W (IRR = 0.89, 95% CI = 0.76–1.03, *P*‐value = 0.133).

For WSAS, BUP‐XR was associated with improved functioning at week 12 (mean difference −3.79, 95% CI = − 6.82 to −0.77, *P*‐value = 0.014). Lower impairment was associated with more opioid‐free days in the outcome model (IRR = 0.99 per one‐point increase in WSAS, 95% CI = 0.98–0.99, *P*‐value = 0.001). The treatment × mediator interaction term was >1, but small (IRR = 1.01, 95% CI = 1.00–1.02, *P*‐value = 0.004), indicating weak synergistic interaction.

### Four‐way decomposition

The four‐way decomposition (Table 3) showed statistically significant total effects of BUP‐XR on opioid‐free days across all four mediator models: CEQ‐F (IRR = 1.17, 95% CI = 1.07–1.29, *P*‐value = 0.001), VAS‐N (IRR = 1.13, 95% CI = 1.04–1.23, *P*‐value = 0.005), VAS‐W (IRR = 1.18, 95% CI = 1.07–1.29, *P*‐value <0.001) and WSAS (IRR = 1.10, 95% CI = 1.02–1.19, *P*‐value = 0.015). E‐values for the confidence limits closest to the null ranged from 1.43 to 1.63, suggesting that unmeasured confounding would need to be moderately associated with both treatment and outcome to explain away the observed total effects.

**TABLE 3:** Four‐way decomposition by mediator.

Model	Component	IRR	95% CI	*P*‐value
CEQ‐F	Total	1.17	1.07–1.29	0.001
	PIE	1.02	0.99–1.05	0.178
	INTmed	0.98	0.95–1.01	0.174
Present	CDE	1.27	1.11–1.44	<0.001
	INTref	0.93	0.87–0.99	0.024
Absent	CDE	1.05	0.96–1.15	0.297
	INTref	1.12	1.02–1.23	0.022
VAS‐N	Total	1.13	1.04–1.23	0.005
	PIE	1.05	1.01–1.10	0.015
	INTmed	0.95	0.91–1.00	0.030
Present	CDE	1.36	1.16–1.60	<0.001
	INTref	0.83	0.74–0.93	0.002
Absent	CDE	1.03	0.96–1.11	0.448
	INTref	1.10	1.02–1.18	0.011
VAS‐W	Total	1.18	1.07–1.29	<0.001
	PIE	1.02	0.98–1.07	0.257
	INTmed	0.97	0.94–1.01	0.200
Present	CDE	1.25	1.09–1.42	0.001
	INTref	0.94	0.87–1.02	0.149
Absent	CDE	1.10	0.99–1.23	0.077
	INTref	1.07	0.98–1.17	0.145
WSAS	Total	1.10	1.02–1.19	0.015
	PIE	1.05	0.99–1.11	0.085
	INTmed	0.95	0.90–1.01	0.106
	CDE	1.10	1.02–1.19	0.016
	INTref	0.99	0.95–1.04	0.809

Abbreviations: CDE, controlled direct effect; CEQ‐F, Craving Experiences Questionnaire‐Frequency version; INTref, reference interaction; INTmed, mediated interaction; IRR, adjusted incidence rate ratio; PIE, pure indirect effect; VAS‐N, ‘need’ for opioids; VAS‐W, ‘want’ for opioids; WSAS, Work and Social Adjustment Scale.

VAS‐N provided the clearest evidence that the treatment effect operated partly through the mediator. The pure indirect effect was statistically significant (PIE = 1.05, 95% CI = 1.01–1.10, *P*‐value = 0.015), indicating that reductions in perceived opioid ‘need’ contributed to the treatment effect. The mediated‐interaction component was also statistically significant (INTmed = 0.95, 95% CI = 0.91–1.00, *P*‐value = 0.030), indicating antagonistic mediated interaction on the multiplicative scale. When craving was present, the controlled direct effect was substantial (CDE = 1.36, 95% CI = 1.16–1.60, *P*‐value <0.001) and the reference interaction component indicated antagonism (INTref = 0.83, 95% CI = 0.74–0.93, *P*‐value = 0.002). When craving was absent, the controlled direct effect was close to the null (CDE = 1.03, 95% CI = 0.96–1.11, *P*‐value = 0.448), while the reference interaction indicated weak synergy (INTref = 1.10, 95% CI = 1.02–1.18, *P*‐value = 0.011).

For CEQ‐F, the pure indirect effect was small and not statistically significant (PIE = 1.02, 95% CI = 0.99–1.05, *P*‐value = 0.178), and the mediated‐interaction component was also not statistically significant (INTmed = 0.98, 95% CI = 0.95–1.01, *P*‐value = 0.174). When craving was present, the controlled direct effect was substantial (CDE = 1.27, 95% CI = 1.11–1.44, *P*‐value <0.001) and the reference interaction indicated antagonism (INTref = 0.93, 95% CI = 0.87–0.99, *P*‐value = 0.024). When craving was absent, the controlled direct effect was smaller and not statistically significant (CDE = 1.05, 95% CI = 0.96–1.15, *P*‐value = 0.297), whereas the reference interaction indicated weak synergy (INTref = 1.12, 95% CI = 1.02–1.23, *P*‐value = 0.022). Overall, CEQ‐F did not appear to meaningfully transmit the treatment effect, which was expressed mainly through the controlled direct pathway.

VAS‐W showed the weakest evidence of mediation among the craving measures. Both the pure indirect and mediated‐interaction components were small and not statistically significant (PIE = 1.02, 95% CI = 0.98–1.07, *P*‐value = 0.257; INTmed = 0.97, 95% CI = 0.94–1.01, *P*‐value = 0.200). When craving was present, the controlled direct effect was substantial (CDE = 1.25, 95% CI = 1.09–1.42, *P*‐value = 0.001), but the reference interaction was not statistically significant (INTref = 0.94, 95% CI = 0.87–1.02, *P*‐value = 0.149). When craving was absent, neither the controlled direct effect (CDE = 1.10, 95% CI = 0.99–1.23, *P*‐value = 0.077) nor the reference interaction (INTref = 1.07, 95% CI = 0.98–1.17, *P*‐value = 0.145) was statistically significant. For WSAS, the pure indirect and mediated‐interaction components were both close to 1 and not statistically significant (PIE = 1.05, 95% CI = 0.99–1.11, *P*‐value = 0.085; INTmed = 0.95, 95% CI = 0.90–1.01, *P*‐value = 0.106). The total effect was expressed primarily through the controlled direct effect (CDE = 1.10, 95% CI = 1.02–1.19, *P*‐value = 0.016), while the reference‐interaction component was close to the null and not statistically significant (INTref = 0.99, 95% CI = 0.95–1.04, *P*‐value = 0.809), indicating little evidence that personal functioning modified the treatment–outcome relationship.

### Sensitivity analyses

Across deterministic (Table 4) analyses, the direction and magnitude of the principal mediation findings for the craving variables were materially unchanged. VAS‐N consistently showed the strongest evidence of mediation across all analytical approaches. Estimates for VAS‐W and CEQ‐F remained directionally consistent, but were less precise in some sensitivity analyses, while WSAS findings remained comparatively weak and somewhat sensitive to assumptions regarding missingness.

**TABLE 4:** Four‐way decomposition analysis addressing missing craving mediator data.

Model	Effect	Best‐case			Worst‐case		
		IRR	95% CI	*P*‐value	IRR	95% CI	*P*‐value
CEQ‐F *n* = 314	Total	1.19	1.08–1.32	<0.001	1.24	1.11–1.37	<0.001
	PIE	1.00	0.99–1.02	0.596	1.05	1.01–1.10	0.015
	INTmed	0.99	0.98–1.01	0.586	0.95	0.91–1.00	0.030
	CEQ‐F present						
	CDE	1.29	1.13–1.48	<0.001	1.30	1.14–1.49	<0.001
	INTref	0.93	0.85–1.01	0.084	0.94	0.90–0.99	0.013
	CEQ‐F absent						
	CDE	1.13	1.00–1.27	0.041	1.05	0.95–1.15	0.337
	INTref	1.06	0.99–1.13	0.079	1.17	1.05–1.32	0.006
VAS‐N *n* = 314	Total	1.15	1.05–1.26	0.002	1.22	1.10–1.35	<0.001
	PIE	1.03	1.00–1.05	0.019	1.11	1.04–1.18	0.001
	INTmed	0.97	0.94–1.00	0.038	0.92	0.86–0.98	0.007
	VAS‐N present						
	CDE	1.39	1.18–1.63	<0.001	1.37	1.16–1.62	<0.001
	INTref	0.83	0.73–0.94	0.005	0.88	0.80–0.96	0.003
	VAS‐N absent						
	CDE	1.08	0.99–1.19	0.080	1.03	0.95–1.12	0.461
	INTref	1.06	1.01–1.11	0.016	1.17	1.05–1.30	0.004
VAS‐W *n* = 314	Total	1.20	1.09–1.33	<0.001	1.23	1.11–1.37	<0.001
	PIE	1.01	0.99–1.02	0.572	1.06	1.00–1.12	0.042
	INTmed	0.99	0.97–1.01	0.386	0.96	0.91–1.01	0.089
	VAS‐W present						
	CDE	1.28	1.11–1.47	0.001	1.28	1.12–1.47	<0.001
	INTref	0.95	0.86–1.04	0.265	0.95	0.91–1.00	0.066
	VAS‐W absent						
	CDE	1.17	1.03–1.32	0.014	1.10	0.98–1.23	0.108
	INTref	1.04	0.97–1.10	0.271	1.11	0.99–1.25	0.065

*Note*: Best‐case, imputes missing craving mediator data as absent.

Worst‐case, imputes missing craving mediator data as present.

Abbreviations: CDE, controlled direct effect; CEQ‐F, Craving Experiences Questionnaire‐Frequency version; INTref, reference interaction; INTmed, mediated interaction; IRR, adjusted incidence rate ratio; PIE, pure indirect effect; VAS‐N, ‘need’ for opioids; VAS‐W, ‘want’ for opioids.

Under deterministic sensitivity assumptions, total effects remained statistically significant across all models. For VAS‐N, all decomposition components remained stable across sensitivity scenarios. CEQ‐F estimates showed greater variability, with some indirect and interaction components reaching statistical significance under the worst‐case assumption. For VAS‐W, component estimates remained small and directionally consistent across scenarios.

For WSAS sensitivity analyses using mean substitution for missing mediator values identified small, but statistically significant indirect and interaction components (PIE = 1.08, 95% CI = 1.02–1.15, *P*‐value = 0.010; INTmed = 0.93, 95% CI = 0.87–0.98, *P*‐value = 0.010), although these effects were modest in magnitude.

In the MICE analysis (Table S1), estimates generally lay between the corresponding best‐case and worst‐case deterministic estimates, with no substantive change in interpretation.

### Treatment‐effect modification

The treatment‐effect modification analysis (Table 5) showed that the total effect of BUP‐XR on opioid‐free days was statistically significant among participants with higher baseline OUD severity (IRR = 1.35, 95% CI = 1.01–1.80, *P*‐value = 0.040) and among those with a longer treatment episode before enrolment (IRR = 1.12, 95% CI = 1.01–1.23, *P*‐value = 0.026). Across these subgroup analyses, VAS‐N remained the only mediator to show any evidence consistent with partial mediation, in keeping with the main analysis. WSAS components were generally close to 1 and not statistically significant.

**TABLE 5:** Stratified four‐way decomposition analysis by baseline OUD severity and treatment duration.

Model	Effect	OUD severity			Treatment duration		
		IRR	95% CI	*P*‐value	IRR	95% CI	*P*‐value
CEQ‐F	Total	1.41	1.06–1.88	0.018	1.14	1.03–1.26	0.014
	PIE	1.09	0.94–1.26	0.256	1.02	0.98–1.05	0.364
	INTmed	0.91	0.79–1.06	0.243	0.99	0.95–1.02	0.382
Present	CDE	1.48	1.12–1.97	0.007	1.22	1.05–1.43	0.011
	INTref	0.96	0.88–1.04	0.299	0.93	0.86–1.01	0.068
Absent	CDE	0.97	0.68–1.38	0.868	1.04	0.95–1.14	0.370
	INTref	1.46	0.97–2.20	0.070	1.09	1.00–1.20	0.060
VAS‐N	Total	1.35	1.01–1.80	0.040	1.12	1.01–1.23	0.026
	PIE	1.19	1.02–1.39	0.023	1.02	0.98–1.06	0.257
	INTmed	0.86	0.72–1.03	0.109	0.98	0.94–1.02	0.306
Present	CDE	1.49	1.08–2.04	0.014	1.32	1.07–1.63	0.010
	INTref	0.88	0.74–1.05	0.148	0.84	0.72–0.99	0.034
Absent	CDE	1.03	0.79–1.34	0.809	1.04	0.96–1.13	0.326
	INTref	1.27	0.94–1.72	0.117	1.07	0.99–1.15	0.071
VAS‐W	Total	1.41	1.08–1.85	0.012	1.14	1.02–1.26	0.017
	PIE	1.05	0.87–1.27	0.609	1.03	0.98–1.08	0.206
	INTmed	0.96	0.81–1.13	0.623	0.97	0.93–1.02	0.231
Present	CDE	1.45	1.10–1.91	0.008	1.23	1.04–1.45	0.015
	INTref	0.97	0.85–1.10	0.623	0.92	0.85–1.01	0.074
Absent	CDE	1.26	0.80–2.01	0.319	1.04	0.95–1.13	0.380
	INTref	1.11	0.75–1.63	0.600	1.09	0.99–1.20	0.070
WSAS	Total	1.27	0.99–1.63	0.059	1.07	0.99–1.16	0.104
	PIE	1.08	0.95–1.22	0.225	1.03	0.98–1.09	0.211
	INTmed	0.93	0.82–1.05	0.231	0.97	0.91–1.02	0.234
	CDE	1.30	1.02–1.64	0.031	1.07	0.99–1.16	0.097
	INTref	0.98	0.88–1.09	0.703	1.00	0.96–1.05	0.977

*Note*: OUD severity is moderate‐extremely severe on Clinical Global Impression–Severity scale.

Treatment duration is <28 days versus ≥28 days at study enrolment.

Abbreviations: CDE, controlled direct effect; CEQ‐F, Craving Experiences Questionnaire – Frequency version; INTref, reference interaction; INTmed, mediated interaction; IRR, adjusted incidence rate ratio; OUD, opioid use disorder; PIE, pure indirect effect; VAS‐N, ‘need’ for opioids; VAS‐W, ‘want’ for opioids; WSAS, Work and Social Adjustment Scale.

Among participants with higher baseline OUD severity, both the total effect and the indirect pathway through reduced perceived opioid ‘need’ were statistically significant (total IRR = 1.35, 95% CI = 1.01–1.80, *P*‐value = 0.040; PIE = 1.19, 95% CI = 1.02–1.39, *P*‐value = 0.023). There was no clear evidence of additional interaction‐related effects in this subgroup.

Among participants with a longer treatment episode before enrolment (≥28 days), the total effect remained statistically significant (IRR = 1.12, 95% CI = 1.01–1.23, *P*‐value = 0.026), but the indirect pathway via VAS‐N was not (PIE = 1.02, 95% CI = 0.98–1.06, *P*‐value = 0.257). In this subgroup, the controlled direct effect was the dominant component (CDE = 1.32, 95% CI = 1.07–1.63, *P*‐value = 0.010). The reference‐interaction component indicated weak antagonism (INTref = 0.84, 95% CI = 0.72–0.99, *P*‐value = 0.034), whereas the mediated‐interaction component was not statistically significant (INTmed = 0.98, 95% CI = 0.94–1.02, *P*‐value = 0.306). Across both modifier analyses, CEQ‐F and VAS‐W showed little evidence of mediation, with no clear evidence of subgroup‐specific mediation pathways. Overall, the pattern was consistent with the main complete‐case analysis, with perceived opioid ‘need’ again showing the clearest evidence of involvement in the treatment effect.

## DISCUSSION

Our causal mediation analysis offers insight into potential mechanisms through which BUP‐XR treatment effects may operate via changes in craving and personal functioning. We found consistent associations between treatment, candidate mediators and opioid‐free days, although the magnitude and configuration of effects differed across mediators. VAS‐N showed the clearest evidence of mechanistic involvement, with statistically significant indirect and mediated‐interaction components. Findings were broadly consistent across deterministic and MICE sensitivity analyses, and E‐values suggested that unmeasured confounding would need to be at least moderately associated with both treatment and outcome to explain away the observed total effects.

### Comparative evaluation of the craving measures

Taken together, VAS‐N was the only mediator that showed statistically significant indirect and mediated‐interaction components, making it the clearest candidate mechanistic pathway in these analyses. Comparisons across mediators should nevertheless be interpreted cautiously because complete‐case analysis resulted in different analytic samples across models. In the VAS‐N model, participants receiving BUP‐XR achieved approximately 13% more opioid‐free days than those receiving MET/BUP‐SL (IRR = 1.13), and the pure indirect effect (PIE = 1.05) suggested that reduced need‐related craving contributed to the treatment effect.

The mediated interaction (INTmed <1) suggested antagonism on the multiplicative scale, and BUP‐XR remained effective when craving persisted, but its incremental benefit was smaller than expected under multiplicativity when craving was absent. This is consistent with accounts of opioid ‘need’ as an aversive interoceptive signal linked to negative reinforcement [3] and with the pharmacological profile of BUP‐XR as a long‐acting partial agonist that reduces withdrawal‐related states. Our mixed‐methods evaluation of EXPO is also consistent with this interpretation, with participants often describing BUP‐XR as helping them feel ‘normal’ again [33].

The CEQ‐F showed a broadly similar, but attenuated pattern compared with VAS‐N, with smaller and non‐significant mediated components. Because CEQ‐F aggregates imagery, intrusiveness and attentional capture, it may be less sensitive to the interoceptive aspect of craving most directly influenced by BUP‐XR. Subscales might reveal more specific effects, but only total CEQ‐F scores were available in EXPO. Future studies might find CEQ‐F more informative in psychosocial or cue‐focused interventions, where cognitive craving processes may play a larger mechanistic role.

In contrast, VAS‐W showed little evidence of mediation. Although wanting predicted opioid use in the outcome models, the four‐way decomposition indicated that reductions in wanting were not a clear mechanism through which BUP‐XR exerted its benefit. The treatment effect was expressed primarily through the controlled direct effect, suggesting that the benefits of BUP‐XR were not meaningfully transmitted through cue‐reactive wanting as captured by this measure. BUP‐XR would not be expected to abolish all cue‐driven wanting, and psychotherapeutic approaches targeting incentive salience may remain important [34].

These findings are consistent with etiological models that distinguish negative reinforcement (need‐driven use) from incentive salience (cue‐driven wanting). They suggest that BUP‐XR's main mechanistic leverage may lie in suppressing need‐based interoceptive signals rather than substantially altering cue‐reactive motivational processes. This also accords with clinical experience, as individuals with OUD often describe a strong drive to avoid withdrawal and a need for opioids to restore physiological normality. By contrast, people with stimulant use disorder more often describe intense wanting while rejecting the idea that their body needs stimulants. Although comparative empirical data are limited, this clinical contrast may help contextualise the distinction between need‐related and want‐related craving observed here.

### Personal functioning

BUP‐XR was associated with improved functioning as measured by the WSAS, and lower impairment predicted more opioid‐free days. However, in the four‐way decomposition, indirect and mediated‐interaction components were small and not statistically significant, and the controlled direct effect accounted for most of the total effect. These findings suggest that personal functioning contributed less clearly to outcome than the craving mediators and any mediating influence was modest relative to the direct treatment effect.

Interpretation of WSAS as a mediator is further limited by greater missingness than for the craving measures, which reduced precision and may have obscured modest mediated effects. WSAS may, therefore, be better viewed as a clinically important correlate of treatment response than as a distinct causal pathway. Nevertheless, because improvement in personal functioning is a core therapeutic goal, its inclusion as an exploratory mediator remained important.

### Treatment‐effect modification

Treatment‐effect modification analyses varied according to baseline OUD severity and duration of the current treatment episode before enrolment, with larger total effects among participants with greater baseline severity and among those who had already been in treatment for 28 days or more. Across subgroups, VAS‐N remained the only mediator to show any evidence consistent with partial mediation in the subgroup analyses. CEQ‐F and VAS‐W again showed limited evidence of mediation, and WSAS did not appear to transmit treatment effects in any subgroup. Interaction components were generally small and imprecise and did not materially alter the main mechanistic interpretation. Simulation studies suggest that treatment‐effect modification estimates can be sensitive to moderator–confounder interactions, and modest misspecification may bias subgroup estimates even when main effects remain stable [35]. Consistent with this, some interaction components varied across subgroup analyses, but these were small and unstable and did not alter the overall interpretation. The relative stability of the total effect across subgroups suggests that need‐related craving reduction was more informative as a candidate mechanism than baseline severity or treatment duration were as effect modifiers.

### Strengths and limitations

Study strengths include the randomised design and sequential assessment of treatment, mediator and outcome, which support causal interpretation [30]. A further strength was the use of VanderWeele's four‐way decomposition, which allowed mediation and treatment–mediator interaction to be estimated separately and, therefore, provided a more differentiated account of treatment mechanisms. By contrast, classical approaches such as Baron and Kenny [36] do not explicitly distinguish mediation from treatment–mediator interaction and might, therefore, have suggested broader evidence of partial mediation across the craving measures.

Several limitations should be noted. First, participants entering from MET represented a selected subgroup rather than the full population receiving this treatment, which may limit generalisability of the comparator findings. Comparator oral treatment doses reflected routine UK clinical practice among participants willing and eligible to enter a trial involving potential transition to BUP‐XR, but may not represent higher‐dose maintenance populations.

Second, comparisons between mediators should be interpreted cautiously because complete‐case samples differed across models and the mediators were not constructed equivalently. Craving measures summarised responses across weeks 4, 8 and 12, whereas WSAS was assessed at week 12 only. Thereforem we did not model the dynamic, and potentially reciprocal, relationships between craving and opioid use over time. More complex longitudinal approaches may be better suited to capturing such processes [37], but these introduce additional assumptions and were beyond the scope of the present analysis, which was intended to provide a parsimonious summary of mediation during early treatment.

Third, approximately one‐third of participants did not contribute complete follow‐up outcome data during weeks 13 to 24, largely reflecting treatment discontinuation rather than administrative loss to follow‐up. Because post‐discontinuation research assessments were not routinely collected, the mediation analyses necessarily relied on observed follow‐up data contributed during study participation. We addressed intermittent missing mediator data using deterministic and multiple imputation sensitivity analyses and incorporated observed follow‐up time into the outcome models using an exposure variable. Nevertheless, differential treatment retention between groups may still have influenced mediator and outcome estimates and should be considered when interpreting the findings.

Fourth, mediator measures were self‐reported and, therefore, subject to recall and response bias, although opioid‐use outcomes were corroborated by UDS. Ecological momentary assessment (EMA) can offer greater temporal precision [38], but its feasibility alongside an extended‐release treatment designed to simplify daily management is uncertain. EMA may help future studies identify optimal measurement windows, although its ability to capture elaborated cognitions underpinning craving may be more limited [39].

### Implications for practice and research

Our findings suggest that the therapeutic benefit of BUP‐XR arises primarily through direct pathways, with additional benefit associated with suppression of need‐based craving. Perceived opioid ‘need’ may therefore represent an important proximal mechanistic endpoint in trials of OUD pharmacotherapies. Given its low burden, scalability and clinical interpretability, future trials of medications and behavioural interventions for OUD should consider including a brief VAS‐based assessment of opioid ‘need’. This could sit alongside a core outcome set for OUD treatment research [40]. Standardising this measure may facilitate clearer mechanistic inference and improve interpretability of treatment effects across trials.

## CONCLUSION

In this pre‐registered exploratory analysis, BUP‐XR appeared to increase opioid‐free days primarily through direct pathways, with additional benefit associated with reduced perceived need for opioids. This aspect of craving showed the clearest evidence of mechanistic involvement, and improvements in personal functioning appeared to occur alongside, rather than clearly mediating, treatment response. These findings illustrate the value of four‐way decomposition approaches for clarifying mechanisms of action and support further development of mechanistically informed studies of OUD treatment.

## AUTHOR CONTRIBUTIONS

**John Marsden:** Conceptualization; writing—original draft; methodology; visualization; formal analysis; data curation; project administration; resources. **Mike Kelleher:** Investigation; funding acquisition; writing—review and editing; project administration; resources. **Fiona Cowden:** Investigation; writing—review and editing; project administration; resources. **Edward Day:** Investigation; writing—review and editing; resources; project administration. **Jonathan Dewhurst:** Investigation; writing—review and editing; project administration; resources. **Rachel Evans:** Investigation; validation; writing—review and editing; software; formal analysis. **Eilish Gilvarry:** Investigation; writing—review and editing; project administration; resources. **Natalie Lowry:** Investigation; writing—review and editing; project administration. **Luke Mitcheson:** Investigation; writing—review and editing; resources; project administration. **Robert Murray:** Investigation; writing—review and editing. **Sophie Quarshie:** Investigation; writing—review and editing; resources; project administration. **Zoë Hoare:** Conceptualization; investigation; methodology; validation; writing—review and editing; software; formal analysis; supervision; resources; data curation.

## DECLARATION OF INTERESTS

J.M., M.K., L.M., E.G. and Z.H. declare collaborative research grant funding for the EXPO study from Indivior. J.M. declares research grant funding from Beckley PsyTech (phase 2a trial of 5‐MeO‐DMT for AUD); from the National Institute for Health Research (NIHR) for a phase 2b trial of medications for cocaine use disorder (MEDCO); and a phase 3 trial of recovery support for substance use disorders (SUPPORT). He is the senior scientific advisor for the Office for Health Improvement and Disparities, English Department of Health and Social Care and a clinical academic consultant for the US National Institute on Drug Abuse, Centre for the Clinical Trials Network. J.M. declares honoraria and travel support from PCM Scientific, OPEN Health and Indivior to contribute to scientific and educational meetings. M.K. declares research grant funding from Beckley PsyTech (phase 2a trial of 5‐MeO‐DMT for AUD) and from the NIHR for the MEDCO and SUPPORT projects. He is the national clinical advisor for the Office for Health Improvement and Disparities, English Department of Health and Social Care. E.D. declares research grant funding from the NIHR for SUPPORT and a phase 3 trial of facilitated access to mutual aid (FAMA). E.D. is the UK Government National Recovery Champion, seconded part‐time to the Home Office. E.G. declares research grant funding from the NIHR for the SUPPORT and MEDCO projects. L.M. declares research grant funding from the NIHR for a realist evaluation of services for people with co‐occurring mental health and substance use disorders, a study of ketamine for AUD and for the FAMA, SUPPORT and MEDCO projects. He has a clinical psychology secondment at the Office for Health Improvement and Disparities, English Department of Health and Social Care. S.Q. declares research grant funding from the NIHR for the SUPPORT and MEDCO projects. All other authors have no competing interests to declare.

The funder of the study (Indivior) provided comments on the manuscript for accuracy, but had no role in study design, data collection, data analysis, data interpretation, or writing of the report. For the purpose of open access, the author has applied a Creative Commons attribution (CC BY) licence to any author accepted manuscript version arising.

## Supporting information


**Table S1:** Mediator and outcome models, sensitivity analysis for missing baseline and mediator variables using best‐case, worst‐case assumption and multiple imputation (*n* = 314).

## Data Availability

De‐identified patient data will not be shared unless a data‐access proposal submitted to the corresponding author by researchers from non‐commercial organisations is approved by the sponsor and research partners, and, where required, by the Health Research Authority, as having sufficient scientific merit and value within the study publication plan.
