# Supplementary material for: Craving and personal functioning as mediators of extended‐release buprenorphine efficacy: A four‐way decomposition analysis from a randomised trial
Source: Addiction. 2026 Jul 1;121(11):3010–24. doi: 10.1111/add.70521 (PMC13677813; doi:10.1111/add.70521)
Supplement: Supplementary file 1 [file ADD-121-3010-s001.docx]

**Table S1**: Mediator and outcome models, sensitivity analysis for missing baseline and mediator variables using best-case, worst-case assumption and multiple imputation (n=314)

|  |  |  | **Best** |  | **Worst** |  | **MI** |  |
| --- | --- | --- | --- | --- | --- | --- | --- | --- |
| **Measure** | **Model** | **Analysis** | **Effect** | **95% CI** | **Effect** | **95% CI** | **Effect** | **95% CI** |
| CEQ-F | OR | Group | 1.17 | 0.72-1.92 | 2.60 | 1.54-4.41 | 1.81 | 1.02-3.22 |
|  |  | Baseline heroin | 0.40 | 0.23-0.70 | 0.50 | 0.27-0.92 | 0.41 | 0.21-0.81 |
|  |  | Baseline craving | 0.98 | 0.97-0.99 | 0.98 | 0.96-0.99 | 0.97 | 0.96-0.99 |
|  | IRR | Group | 1.27 | 1.13-1.43 | 1.28 | 1.14-1.44 | 1.28 | 1.12-1.46 |
|  |  | CEQ-F | 1.10 | 0.97-1.25 | 1.26 | 1.12-1.42 | 1.18 | 0.98-1.42 |
|  |  | Group x CEQ-F | 0.88 | 0.77-1.00 | 0.81 | 0.71-0.92 | 0.84 | 0.67-1.05 |
|  |  | Baseline heroin | 0.95 | 0.88-1.03 | 0.97 | 0.90-1.04 | 0.96 | 0.84-1.10 |
|  |  | Baseline craving | 1.00 | 1.00-1.00 | 1.00 | 1.00-1.00 | 1.00 | 1.00-1.00 |
| VAS-N | OR | Group | 2.32 | 1.37-3.91 | 3.94 | 2.40-6.48 | 3.13 | 1.78-5.51 |
|  |  | Baseline heroin | 0.27 | 0.15-0.49 | 0.49 | 0.28-0.85 | 0.35 | 0.19-0.66 |
|  |  | Baseline craving | 0.99 | 0.98-1.00 | 0.99 | 0.98-1.00 | 0.99 | 0.98-1.00 |
|  | IRR | Group | 1.40 | 1.19-1.65 | 1.37 | 1.18-1.60 | 1.41 | 1.18-1.67 |
|  |  | VAS-N | 1.29 | 1.12-1.50 | 1.40 | 1.21-1.62 | 1.34 | 1.13-1.57 |
|  |  | Group x VAS-N | 0.77 | 0.65-0.91 | 0.75 | 0.63-0.88 | 0.75 | 0.60-0.93 |
|  |  | Baseline heroin | 0.97 | 0.90-1.05 | 0.98 | 0.90-1.05 | 0.97 | 0.86-1.10 |
|  |  | Baseline craving | 1.00 | 1.00-1.00 | 1.00 | 1.00-1.00 | 1.00 | 1.00-1.00 |
| VAS-W | OR | Group | 1.58 | 0.95-2.63 | 3.39 | 2.00-5.74 | 2.51 | 1.40-4.50 |
|  |  | Baseline heroin | 0.36 | 0.20-0.65 | 0.47 | 0.25-0.86 | 0.39 | 0.20-0.76 |
|  |  | Baseline craving | 0.98 | 0.97-0.99 | 0.98 | 0.97-0.99 | 0.98 | 0.97-0.99 |
|  | IRR | Group | 2.51 | 1.4-4.5.00 | 1.26 | 1.12-1.42 | 1.26 | 1.09-1.46 |
|  |  | VAS-W | 0.39 | 0.20-0.76 | 1.20 | 1.04-1.39 | 1.13 | 0.93-1.36 |
|  |  | Group x VAS-W | 0.98 | 0.97-0.99 | 0.86 | 0.74-0.99 | 0.88 | 0.70-1.10 |
|  |  | Baseline heroin | 0.98 | 0.97-0.99 | 0.96 | 0.89-1.04 | 0.95 | 0.83-1.10 |
|  |  | Baseline craving | 2.90 | 1.85-4.54 | 1.00 | 1.00-1.00 | 1.00 | 1.00-1.00 |
| **Measure** | **Model** | **Analysis** |  |  | **Mean** |  | **MI** |  |
| WSAS | Coef. | Group |  |  | -5.65 | -7.7- -3.59 | -4.62 | -7.65- -1.6 |
|  |  | Baseline heroin |  |  | 1.84 | -0.36-4.03 | 2.45 | -0.56-5.45 |
|  |  | Baseline WSAS |  |  | 0.27 | 0.19-0.35 | 0.40 | 0.28-0.52 |
|  | IRR | Group |  |  | 1.01 | 0.92-1.11 | 1.06 | 0.92-1.23 |
|  |  | WSAS |  |  | 0.99 | 0.98-0.99 | 0.99 | 0.98-1.00 |
|  |  | Group x WSAS |  |  | 1.01 | 1.00-1.02 | 1.01 | 1.00-1.02 |
|  |  | Baseline heroin |  |  | 0.89 | 0.83-0.95 | 0.89 | 0.79-1.00 |
|  |  | Baseline WSAS |  |  | 1.00 | 0.99-1.00 | 1.00 | 0.99-1.00 |

Group (0, MET/BUP-SL; 1, BUP-XR),

CI, Confidence Interval; CEQ-F, Craving Experiences Questionnaire – Frequency version;

IRR, adjusted Incidence Rate Ratio; MI, multiple imputation - logistic regression imputation was used for CEQ-F, VAS-N, and VAS-W, predictive mean matching for the WSAS; OR, adjusted odds ratio;

VAS-N, ‘need’ for opioids; VAS-W, ‘want’ for opioids; WSAS, Work and Social Adjustment Scale (for OUD).

Best-case, imputes missing craving mediator data as absent; Worst-case, imputes missing craving mediator data as present.

OR and Coef. rows show adjusted mediator models for craving (logistic regression) and personal functioning (linear regression). IRR rows show adjusted negative binomial outcome models for opioid-free days including treatment group, mediator, baseline covariates, and the treatment × mediator interaction.
